# The Randomized Freeway Stent Study: Drug-Eluting Balloons Outperform Standard Balloon Angioplasty for Postdilatation of Nitinol Stents in the SFA and PI Segment

**DOI:** 10.1007/s00270-019-02309-3

**Published:** 2019-08-20

**Authors:** Josef Tacke, Stephan Müller-Hülsbeck, Henrik Schröder, Johannes Lammer, Karl Schürmann, Walter Gross-Fengels, Roman Fischbach, Jochen Textor, Lothar Boguth, Christian Loewe, Hannes Häuser, Manfred Gschwendtner, Gunnar Tepe, Rembert Pogge von Strandmann, Stefanie Stahnke, Johannes Dambach, Klaus Hausegger

**Affiliations:** 1 Institut für Diagnostische und Interventionelle Radiologie und Neuroradiologie, Klinikum Passau, Innstraße 76, 94036 Passau, Germany; 2 Radiologie, Diakonissenkrankenhaus Flensburg, Knuthstraße 1, 24939 Flensburg, Germany; 3 Jüdisches Krankenhaus Berlin, Gemeinschaftspraxis für Radiologie, Neuroradiologie & Zentrum für Minimal Invasive Therapie am Jüdischen Krankenhaus Berlin, Heinz-Galinski-Str. 1, 13347 Berlin, Germany; 4 Kardiovaskuläre und Interventionelle Radiologie, Medizinische Universität Wien, Währinger Gürtel 18-20, 1090 Vienna, Austria; 5 Institut für Diagnostische und Interventionelle Radiologie St.-Johannes-Hospital Dortmund, Johannesstraße 9-17, 44137 Dortmund, Germany; 6 Radiologie und Nuklearmedizin, Asklepios Klinik Harburg, Eißendorfer Pferdeweg 52, 21075 Hamburg, Germany; 7 Radiologie, Neuroradiologie und Nuklearmedizin, Asklepios Klinik Altona, Paul-Ehrlich-Str. 1, 22763 Hamburg, Germany; 8 Abteilung für Radiologie Gemeinschaftskrankenhaus Bonn, St. Elisabeth/St. Petrus/St. Johannes gGmbH, Bonner Talweg 4-6, 53113 Bonn, Germany; 9 Institut für Diagnostische und Interventionelle Radiologie, Klinikum Idar-Oberstein GmbH, Dr.-Ottmar-Kohler Str. 2, 55743 Idar-Oberstein, Germany; 10 Klinik für Diagnostische und Interventionelle Radiologie, Klinikum St. Elisabeth Straubing GmbH, St.-Elisabeth-Str. 23, 94315 Straubing, Germany; 11 Institut für Diagnostische und Interventionelle Radiologie, KH Elisabethinen Linz, Fadingerstrasse 1, 4010 Linz, Austria; 12 Radiologie, Klinikum Rosenheim, Pettenkoferstr. 10, 83022 Rosenheim, Germany; 13 Eurocor Tech GmbH, In den Dauen 6a, 53117 Bonn, Germany; 14 Institut für Diagnostische und Interventionelle Radiologie, Klinikum Klagenfurt am Wörthersee, Feschnigstraße 11, 9020 Klagenfurt, Austria

**Keywords:** Drug-eluting balloon, SFA, Nitinol stent, Paclitaxel, PAD

## Abstract

**Purpose:**

The prospective randomized multicenter Freeway study evaluated the possible hemodynamic and clinical benefits of primary stent insertion followed by percutaneous transluminal angioplasty (PTA) with drug-eluting balloons (DEB) over post-stent insertion PTA with standard balloons in the treatment of symptomatic femoropopliteal arteriosclerotic lesions.

**Methods:**

In total, 204 patients in 13 centers in Germany and Austria were enrolled and randomized to primary stenting followed by either FREEWAY™ drug-eluting balloon or standard PTA balloon angioplasty. The primary endpoint was the rate of clinically driven target lesion revascularization (TLR) at 6 months; the secondary endpoints include TLR rate at 12 months and primary patency, shift in Rutherford classification, ankle–brachial index (ABI) and major adverse events (MAE) at 6 and 12 months. Lesion characteristics and vessel patency were analyzed by an independent and blinded corelab.

**Results:**

At 6-month and 12-month follow-up, TLR rate was lower in the DEB arm compared to standard PTA but did not reach statistical significance (4.1% vs. 9.0% *p* = 0.234 and 7.9% vs. 17.7% *p* = 0.064, respectively). Primary patency was significantly better for patients treated with the DEB at 6 months (90.3% vs. 69.8% *p* = 0.001) and 12 months (77.4% vs. 61.0% *p* = 0.027). Improvement in Rutherford classifications was likewise significantly better for patients in the DEB group at 6 (94.9% vs. 84.3% *p* = 0.027) and 12 months (95.5% vs. 79.9% *p* = 0.003). The percentage of patients with an improved ABI of 1.0–1.2 was significantly higher in the DEB group compared to the PTA group at 6 months (55.3% vs. 35.3%; *p* = 0.015) but without significant difference at 12 months (48.2% vs. 32.9%; *p* = 0.055). At 6 months, rate of major adverse events (MAE) was 1% in both arms, and at 12 months 2.2% for the DEB and 3.8% for the PTA group.

**Conclusion:**

The Freeway Stent Study shows that the usage of DEB as a restenosis prophylaxis seems to be safe and feasible. The 12-month follow-up results give a clear sign in favor of the DEB group.

## Introduction

Primary stenting is the recommended therapy to revascularize stenotic or occlusive arteriosclerotic lesion of the femoropopliteal segment [1]. Several studies examined the effectiveness of different treatment methods and compared stenting to standard percutaneous transluminal angioplasty (PTA) [2–6] or PTA with drug-eluting balloons (DEB) with standard balloon angioplasty [7–12]. Self-expanding nitinol stents have shown equal results to PTA or even advantageous effects in the treatment of longer lesions [13–15]. Experience with drug-eluting stents in the femoropopliteal segment showed that paclitaxel seems to outperform limus-eluting stents, contrary to the experience in the coronary arteries [16–21]. One study that compared paclitaxel-eluting stents and paclitaxel-eluting balloons in femoropopliteal long lesions found equal good performance [22]. PTA with DEB can inhibit the intimal hyperplasia after vessel dilatation and help to prevent lesion restenosis. Several DEB studies showed a beneficial effect of delivering paclitaxel to the vessel wall. Some of these studies allowed no or only provisional stenting or left it to the decision of the physician [7, 9, 11, 12, 23]. The aim of the present study was to test the safety and efficacy of postdilatation with the FREEWAY™ DEB versus a standard balloon in occlusive or stenotic femoropopliteal arteries of symptomatic patients with peripheral arterial disease. The here-tested FREEWAY™ DEB was already examined in the PACUBA trial [24] and the Italian Freeway AV study [25] where it showed very positive results in the treatment of in-stent restenosis (ISR) and hemodialysis patients, respectively.

## Materials and Methods

### Trial Design and Study population

**Fig. 1:**
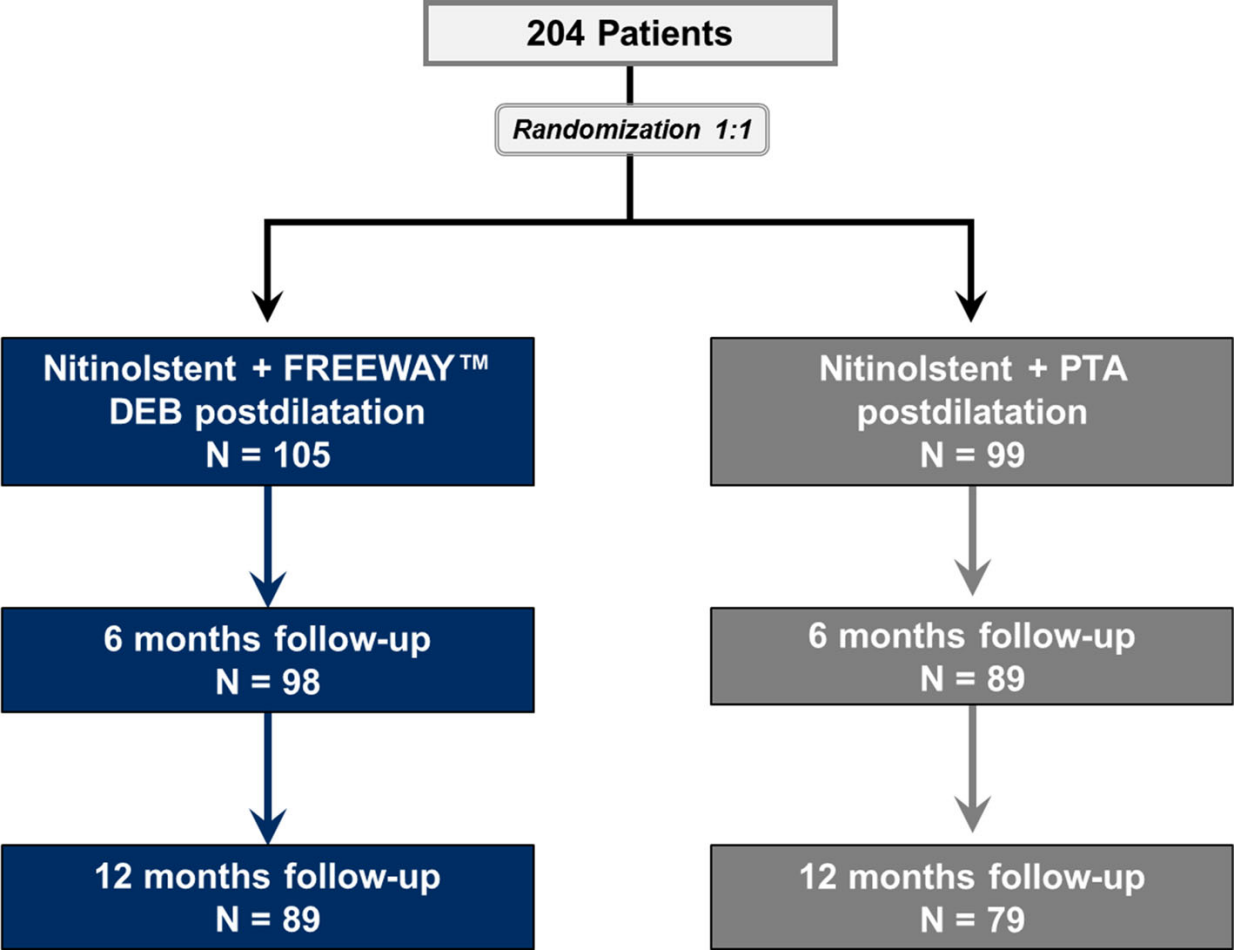
Flowchart of the Freeway Stent Study

The Freeway Stent Study is a prospective, multicenter, open and randomized (1:1) two-arm study. A total of 204 patients in 13 centers in Germany and Austria with stenosis or occlusion in the superficial femoral artery (SFA) and proximal popliteal artery segment (PI segment) were randomized to either arm a) primary nitinol stenting followed by standard PTA or arm b) primary nitinol stenting followed by FREEWAY™ paclitaxel-eluting PTA balloon (Eurocor GmbH) (Fig. 1). The study protocol was approved by the local ethics committees and carried out in accordance with the Good Clinical Practice Guidelines and the Declaration of Helsinki. All patients were intended to give written informed consent. Inclusion criteria were: patients with symptomatic ischemia requiring treatment of SFA or popliteal arteries in one or both legs; Rutherford classification 2–6; lesion length between 4 cm and 15 cm; and a target reference vessel diameter between 4 mm and 7 mm. Exclusion criteria comprised: doubts in the willingness of the patient to allow follow-up examinations; previous bypass surgery or stenting in the target vessel; coexisting of aneurysmal disease of the abdominal aorta, iliac or popliteal arteries; a significant inflow lesion with more than 50 percent stenosis or occlusion, PTA of inflow vessels treated less than 6 months before and absence of at least one patent (less than 50% stenosis) tibioperoneal runoff vessel confirmed by baseline angiography.

The primary endpoint of the study was the rate of clinically driven target lesion revascularization (TLR) at 6 months after the intervention. The secondary endpoints were TLR rate at 12 months, primary patency, shift in Rutherford classification and change in ankle–brachial index (ABI) from baseline to 6 and 12 months, respectively. Further endpoints were major adverse events (MAE) such as death, study-related amputation and thrombosis of target lesion as well as device success.

### Procedure of Treatment, Devices and Medications

Patients were randomized after successful guidewire crossing of the lesion to either a DEB or standard PTA balloon postdilatation of the implanted nitinol stent. The selection of the nitinol stent was up to the decision of the operator. Prior to intervention, patient’s medical history and risk factors were requested. Patient’s clinical status was assessed by Rutherford classification, ankle–brachial index and color duplex ultrasound sonography. Femoral artery access was based on the physician’s decision in a contralateral or ipsilateral manner.

Predilatation of target lesion before stent implantation was optional and performed with a standard PTA balloon. The chosen stent had to cover the length of the target lesion. Vessel reference diameter was estimated by angiography. The stent diameter had to exceed the reference vessel diameter by 1 or 2 mm. If needed, stents could be placed in an overlapping manner with a maximum of two stents (at least 10-mm overlap). Balloons used for post-dilation (standard PTA balloon and FREEWAY™) had to exceed the previously placed stents by a minimum of 3 mm on proximal and distal side to ensure smooth fitting of stent edges to the vessel wall; if necessary a second balloon was used. A repetitive inflation with the same FREEWAY™ balloon was only allowed for postdilatation in exactly that area of the first inflation. For remodeling of reference vessel diameter and sufficient drug delivery, the balloon inflation time was set to 60–120 s. If post-procedure angiography showed residual stenosis > 30%, in both groups inflation with the respective balloon type had to be repeated for 3 min within the already treated area.

Study device was a FREEWAY™ 035 (0.035″ guidewire) drug-eluting balloon (Eurocor Tech GmbH, Bonn, Germany) with a paclitaxel concentration of 3 µg/mm^2^. FREEWAY™ balloons use shellac as drug carrier, which swells in contact with blood and allows a smooth delivery of paclitaxel to the vessel wall. The selection of commercial available self-expanding electro-polished nitinol stents and standard PTA catheters was on the physician’s choice. A list of all used stents is given in Table 1.

**Table 1:** Comparison of used stents in the study arms

Used stent	FREEWAY™ arm *N* = 105 (%)	PTA arm *N* = 99 (%)	*p* value
Absolute/Abbott	2.9	6.7	ns
Epic/Boston Scientific	5.7	6.7	ns
LifeStent/CRBard	11.4	10.5	ns
Misago/Terumo	9.5	4.8	ns
Protégé/EV3	25.7	27.6	ns
S.M.A.R.T./Cordis	31.4	27.9	ns
Other	13.3	10.5	ns

Prescribed medication at discharge comprised 100 mg Aspirin lifelong and 75 mg Clopidogrel for at least 1 month.

### Follow-Up

Patients were asked to come back for follow-up examination after 6 and 12 months. Patient’s clinical status was assessed by Rutherford classification and ankle–brachial index. Potential restenosis was determined by duplex ultrasound (peak velocity ratio (PVR) ≥ 2.4). Major adverse events at the time of follow-up and change in patient’s medication were recorded.

### Corelab Analysis

Data of baseline lesion characteristics and primary patency at 6 and 12 months were analyzed by an independent blinded corelab (coreLab Black Forest GmbH, Bad Krozingen, Germany).

### Definitions

Clinically driven TLR was defined as target lesion revascularization in patients with re-occurrence of ischemic symptoms and target lesion diameter stenosis of > 70% determined by duplex ultrasound. Primary patency was determined as PVR < 2.4 in duplex ultrasound sonography or restenosis < 50% of target vessel diameter by angiographic data. Improvement in Rutherford was determined as a positive shift of ≥ 1 Rutherford classifications from baseline to 6 and 12 months. Degree of vessel calcification was defined by corelab as 0 = none, 1 = mild, 2 = moderate or 3 = severe calcification. Procedural success was defined as less than 30% stenosis at target lesion. Device success was defined as successful delivery and deployment of the study device at the target lesion and successful removal. Patients with more than one MAE were counted only once in the total rate of patients with MAE.

### Statistical Analysis

Sample size calculation was based on expected TLR rates at 6 months. In comparison with the reported TLR rates of previous studies [4, 7, 8], for this study more conservative TLR results were assumed. With a power of 0.80 and an α-error of 0.05, it was calculated that 86 patients per group are needed (Fisher exact test).To take into account a loss of 15% to follow-up, 100 patients were required in each study arm. Continuous data are presented as mean ± standard deviation; hypotheses were tested with unpaired *t* test. Categorical data are presented as absolute patient number and percentage; hypotheses were tested using Fisher’s exact test with two-tailed P value calculation. The statistical significance was determined as *p* < 0.05.

## Results

### Patient Population

A total of 204 patients were enrolled in 13 centers in Germany and Austria and randomized 1:1 for postdilatation of implanted nitinol stents with either FREEWAY™ (*n* = 105) or PTA (*n* = 99) for the treatment of femoropopliteal lesions. Demographic characteristics were evenly distributed among both study arms. Prevalence of risk factors such as diabetes, hypertension, hyperlipidemia and smoking as well as history of cardiovascular diseases showed no significant difference between both groups (Table 2). The proportion of male patients was high in both groups (79.0% FREEWAY™; 76.8% PTA). Baseline clinical status (Rutherford and ABI) was likewise without significant differences between the two groups. The clinical status of the majority of patients at baseline was classified as Rutherford 3 (69.5% FREEWAY™; 68.7% PTA) followed by Rutherford classification 2 (23.8% FREEWAY™; 25.3% PTA) (Table 3). According to their ABI, patients were assigned to defined ABI categories: [> 1.2]; [1.0–1.2]; [0.9–1.0]; [0.8–0.9]; [0.5–0.8]; [< 0.5]. Most patients had an ABI in the range between 0.5 and 0.8 (58.4% FREEWAY™; 69.1% PTA; *p* = 0.14), followed by patients with an ABI lower than 0.5 (21.8% FREEWAY™; 20.6% PTA; *p* = 0.86) (Table 4).

**Table 2:** Baseline patient demographics

	FREEWAY™ DEB + Stent *N* = 105	PTA + Stent *N* = 99	*p* value (ns > 0.05)
Male	79.0%	76.8%	ns
Age	64.7 ± 9.4 years	64.3 ± 9.8 years	ns
Diabetes mellitus	26.7%	26.3%	ns
History of PAD	37.1%	44.4%	ns
History of CAD	24.8%	23.2%	ns
Smoking	88.6%	81.8%	ns
Hyperlipidemia	60.0%	57.6%	ns
Hypertension	75.2%	73.7%	ns

**Table 3:** Rutherford classification of patients at baseline, 6 and 12 months

	FREEWAY™ DEB + Stent			PTA + Stent		
	Baseline *N* = 105	6 Months *N* = 98	12 Months *N* = 88	Baseline *N* = 99	6 Months *N* = 89	12 Months *N* = 79
Rutherford classification						
0	0.0%	80.6%	79.5%	0.0%	68.5%	62.0%
1	0.0%	9.2%	11.4%	0.0%	13.5%	12.7%
2	23.8%	5.1%	5.7%	25.3%	6.7%	7.6%
3	69.5%	3.1%	3.4%	68.7%	7.9%	15.2%
4	1.9%	2.0%	0.0%	1.0%	2.2%	2.5%
5	4.8%	0.0%	0.0%	2.0%	1.1%	0.0%
6	0.0%	0.0%	0.0%	0.0%	0.0%	0.0%
Mean	2.88 ± 0.66	0.37 ± 0.88	0.33 ± 0.47	2.72 ± 0.65	0.65 ± 1.16	0.84 ± 1.23

**Table 4:** ABI at baseline, 6 and 12 months

	FREEWAY™ DEB + Stent			PTA + Stent		
	Baseline *N* = 101 (%)	6 Months *N* = 94 (%)	12 Months *N* = 85 (%)	Baseline *N* = 97 (%)	6 Months *N* = 85 (%)	12 Months *N* = 76 (%)
ABI						
> 1.2	4.0	5.3	11.8	2.1	8.2	6.6
1.0–1.2	1.0	55.3	48.2	2.1	35.3	32.9
0.9–1.0	5.0	21.3	16.5	1.0	20.0	26.3
0.8–0.9	9.9	12.8	9.4	5.2	23.5	6.6
0.5–0.8	58.4	5.3	12.9	69.1	11.8	25.0
< 0.5	21.8	0.0	1.2	20.6	2.4	2.6

### Procedure

Puncture of the ipsilateral femoral artery was chosen in 88.2% (Freeway 89.5%; PTA 86.7%; *p* = 0.66), whereas contralateral femoral access was performed in 11.8% (Freeway 10.5%; PTA 13.3%; *p* = 0.66) of patients. All patients were treated with FREEWAY™ or PTA according to their randomization with envelopes after successful wire crossing of the lesion. Patient lesion characteristics (Table 5) showed no significant differences among both study arms. Lesion locations were predominately in the mid- or distal part of the superficial femoral artery (SFA) with an average lesion length of 7.7 ± 4.2 cm (FREEWAY™) and 8.3 ± 4.1 cm (PTA), respectively. For statistical calculation, longer lesions with overlapping from, for example, distal SFA to PI were always assigned to the location of the proximal beginning of the lesion, in this example to the distal SFA. The mean number of infrapopliteal runoff vessels was 2.26 ± 0.85 (FREEWAY™) and 2.06 ± 0.85 (PTA). The ratio of total occlusions was high in both arms with 63.8% (FREEWAY™) and 63.6% (PTA) (Table 5). Predilatation was done in 73.3% of patients that later received the DEB and 69.7% of patients that received PTA with the standard balloon. Mean parameters of used stents and balloons were similar in both study arms. A significant difference is given in the mean inflation time between DEB and standard balloon (Table 6).

**Table 5:** Lesion characteristics

	FREEWAY™ DEB + Stent *N* = 105	PTA + Stent *N* = 99	*p* value (ns > 0.05)
Lesion location			
SFA prox	6.7%	7.1%	ns
SFA mid	48.6%	43.4%	ns
SFA dist	43.8%	50.5%	ns
PI	1.9%	0.0%	ns
Lesion length	7.7 ± 4.2 cm	8.3 ± 4.1 cm	ns
Diameter stenosis	91.8%	90.9%	ns
Ref. vessel diameter	4.7 ± 0.8 mm	4.6 ± 0.9 mm	ns
Total occlusion	63.8%	63.6%	ns
Vessel calcification	1.45 ± 1.08	1.34 ± 1.04	ns
Infrapopliteal runoff vessels	2.26 ± 0.85	2.06 ± 0.85	ns

Analyzed by an independent blinded corelab

**Table 6:** Procedural data

	FREEWAY™ DEB + Stent *N* = 105	PTA + Stent *N* = 99	*p* value (ns > 0.05)
Predilatation	73.3%	69.7%	ns
Stent			
Length	97.9 ± 37.1 mm	98.9 ± 36.0 mm	ns
Diameter	6.2 ± 0.7 mm	6.3 ± 0.6 mm	ns
Postdilatation study balloon			
Length	86.9 ± 26.7 mm	80.3 ± 26.9 mm	ns
Diameter	5.4 ± 0.6 mm	5.4 ± 0.6 mm	ns
Inflation time study device	107.6 ± 65.0 s	77.3 ± 49.7 s	< 0.001
Inflation pressure study device	9.1 ± 2.0 atm	8.9 ± 1.5 atm	ns
Second study balloon used	54.3%	58.6%	ns
Device success	100%	100%	ns

### Follow-Up

Seventeen of the 204 enrolled patients were lost to 6-month follow-up, and further 19 patients were lost to 12-month follow-up. The TLR rate at 6 months was numerically lower in the FREEWAY™ (4.1%) compared to the PTA group (9.0%) (*p* = 0.234). At 12-month follow-up, the difference in TLR rate between the two arms was close to significance with 7.9% in the FREEWAY™ versus 17.7% in the PTA arm (*p* = 0.064) (Table 7). Primary patency was significantly higher in the FREEWAY™ arm at 6 months (90.3% vs. 69.8% *p* = 0.001) and 12 months (77.4% vs. 61.0% *p* = 0.027) compared to the standard balloon arm, respectively. Likewise significant was the better clinical improvement in one or more Rutherford classifications from baseline to 6 months (94.9% vs. 84.3% *p* = 0.027) and 12 months (95.5% vs. 79.9% *p* = 0.003) for the patients treated with the FREEWAY™ DEB (Table 7). The percentage of patients with an improved ABI of 1.0–1.2 was significantly higher in the DEB group compared to the PTA group at 6 months (55.3% vs. 35.3%; *p* = 0.015) but without statistical significance at 12 months (48.2% vs. 32.9%; *p* = 0.055). The overall safety was very high with only 1.0% and 2.2% of MAE for the DEB and 1.1% and 3.8% of MAE for the PTA group at 6 and 12 months, respectively. No study-related amputations occurred. At 12 months, one patient in every group suffered a thrombosis at target lesion and two patients in the PTA group and one in the DEB group died. All three death cases were not related to the study (Table 8). Causes of death in the PTA arm were myocardial infarction in one patient and renal failure and aspiration pneumonia in the other patient. The death case in the study arm was a diabetic and stroke patient with progressive PAD and CAD who suffered from a wound healing disorder. Cause of death was ischemic cardiomyopathy, TIA and a progressive sepsis.

**Table 7:** Main results at 6- and 12-month follow-up

	FREEWAY™ DEB + Stent (*N*)**	PTA + Stent (*N*)**	*p* value (ns > 0.05)
Primary patency*			
At 6 months	90.3% (93)	69.8% (86)	0.001
At 12 months	77.4% (84)	61.0% (77)	0.027
Clinically driven target lesion revascularization (TLR)			
At 6 months	4.1% (98)	9.0% (89)	0.234
At 12 months	7.9% (89)	17.7% (79)	0.064
Shift in Rutherford from baseline ≥ 1			
At 6 months	94.9% (98)	84.3% (89)	0.027
At 12 months	95.5% (88)	79.9% (79)	0.003

*Analyzed by an independent blinded corelab

**(*N*) = number of evaluated patients

**Table 8:** Major adverse events (MAE) at 6 and 12 months

	FREEWAY™ DEB + Stent *N* = 98*/90**	PTA + Stent *N* = 90*/81**
MAE		
At 6 months	1.0%	1.1%
At 12 months	2.2%	3.8%
Death		
At 6 months	0.0%	1.1%
At 12 months	1.1%	2.5%
Study-related amputation		
At 6 months	0.0%	0.0%
At 12 months	0.0%	0.0%
Thrombosis of target lesion		
At 6 months	1.0%	0.0%
At 12 months	1.0%	1.3%

*(*N*) = number of evaluated patients after 6 months

**(*N*) = number of evaluated patients after 12 months

## Discussion

This study is the first randomized controlled multicenter trial to compare the efficacy and safety of systematic primary nitinol stenting followed by postdilatation with either a DEB or a standard PTA balloon in lesions of the SFA and PI segment. The main findings were a significant advantage in primary patency and a significant better improvement in Rutherford classifications after 6 and 12 months for the patients treated with the DEB. The primary endpoint, the rate of target lesion revascularization at 6 months, was numerically lower in the DEB group but not statistically significant. At 12 months, this difference increased but still did not reach statistical significance. A higher number of patients included in this study could have shown a clear picture for TLR rate. Although the rate of clinically driven TLR at 6 months (primary endpoint) did not show to be statistically significantly better for the DEB group, the clinical and objective hemodynamic data proofed to be significantly better in the DEB group. The here-found advantageous effects of DEB treatment were previously reported for other randomized trials [7–12, 23, 24, 26–28] and non-randomized studies [29–31]. Comparable to this study, systematic stent implantation was performed in the DEBATE SFA trial [23], the RAPID trial [26], the DEBAS study [30] and the BIOLUX 4EVER trial [31]. In contrast to this study, the single-center study of Liistro et al. [23] performed systematic stenting of lesions after previous DEB or PTA dilatation. Their study results showed a positive rate of binary restenosis and TLR for the DEB arm after 12 months. The randomized, multicenter RAPID trial [26] compared a DEB + stent treatment with the stenting alone. The results at 12 months are in favor of the group treated with DEB. Likewise to the Freeway Stent Study, the single-arm, single-center DEBAS study [30] performed DEB dilatation after implantation of nitinol stents. After 12 months, primary patency and freedom from TLR were 94%. The single-arm BIOLUX 4EVER trial [31] performed systematic stenting after previous dilatation with a DEB. As the study has no control arm, the authors compared the results with those of the 4EVER trial [32], which implanted the same stent but performed postdilatation by standard PTA. In this non-DEB trial, patency rates were lower and rate of TLR was higher compared to those reported for the BIOLUX 4EVER trial.

To better understand and compare the outcome of randomized trials, the relative difference (delta Δ) between both study arms is an eligible indicator. By comparing not absolute rates but relative differences between the study arms, it allows for a better comparability and balances the discrepancies in patient population and lesion characteristics between the trials. In the Freeway Stent Study, the observed Δ for primary patency (PP) at 12 months is 16.4% and the Δ for TLR is 9.8%. Those values lie within the range of the aforementioned DEB studies which performed systematic stenting (DEBATE SFA: Δ PP 30.3%, Δ TLR 16.3%; RAPID trial: Δ PP 12.4%, Δ TLR 5.2%; BIOLUX 4EVER trial (compared to 4EVER trial): Δ PP 8.5%, Δ TLR 4.3%). The results of the Freeway Stent Study and the results of comparable studies indicate the advantage to combine the dilatation with a DEB with stenting over stenting alone for the treatment of SFA lesions. Compared to study protocols that ruled only one specific stent model [23, 26, 30, 31], the Freeway Stent Study allowed implantation of different commercially available self-expanding nitinol stents (Table 1). The here-found positive results for the stent + DEB group, independent from a specific stent type, can be regarded as a general proof of concept for a stent plus FREEWAY™ DEB treatment.

Promising results of the here-tested DEB were also reported for studies in other indications. Results of the PACUBA trial [24] showed that ISR patients treated with the FREEWAY™ DEB had a significant higher patency than those who received standard PTA. The authors of the Italian Freeway AV study [25] found a significant longer time to re-intervention for hemodialysis patients treated with a FREEWAY™ DEB compared to a standard PTA treatment. Summing up all available studies, DEBs represent an advantageous treatment for the dilatation of different types of indications in the SFA, in combination with or without a stent.

### Limitations

The follow-up period of 12 months does not allow long-term conclusions on potential compensatory or advantageous effects between both study arms after 1 year. Possible occurrence of restenosis at the lesion site after the period of 12 months lies out of the view of this study.

## Conclusion

This prospective, randomized multicenter trial shows the advantageous hemodynamic and clinical effects of DEB postdilatation of nitinol-stented SFA and proximal popliteal lesions at 12 months after the intervention. Patients treated with a DEB had a numerically lower TLR rate at 6 months; however, this difference was not statistically significant between the groups. Patency rates and improvement in Rutherford classifications at 6 and 12 months and ABI at 6 months were significantly better in the group of patients treated with FREEWAY™ DEB. The observed differences in TLR rate and ABI at 12 months were found to be not statistically significant.
